# Multi-kinase targeted therapy as a promising treatment strategy for ovarian tumors expressing sfRon receptor

**DOI:** 10.18632/genesandcancer.205

**Published:** 2020-07-22

**Authors:** Luyao Wang, Lin Wang, Magdalena Cybula, Ana Luiza Drumond-Bock, Katherine M. Moxley, Magdalena Bieniasz

**Affiliations:** ^1^ Oklahoma Medical Research Foundation, Oklahoma City, OK, USA; ^2^ Department of Obstetrics and Gynecology, Division of Gynecologic Oncology, University of Oklahoma Health Science Center, Oklahoma City, OK, USA

**Keywords:** AD80, ovarian cancer, PDX, sfRon, multi-kinase inhibitor

## Abstract

The sfRon kinase is an important therapeutic target in ovarian cancer that contributes to prominent tumor growth and disease progression. We reasoned that a multi-kinase inhibition of sfRon pathway might be an effective strategy to achieve a sustained anti-tumor response, while simultaneously preventing treatment resistance. We performed a detailed dissection of sfRon signaling *in vitro* and demonstrated that S6K1 is a key component of a multi-kinase targeting strategy in sfRon expressing ovarian tumors. We selected AD80 compound that targets several kinases within sfRon pathway including AKT and S6K1, and compared its efficacy with inhibitors that selectively target either sfRon or PI3 kinase. Using human ovarian xenografts and clinically relevant patient-derived xenografts (PDXs), we demonstrated that *in vivo* treatment with single agent AD80 shows superior efficacy to a standard-care chemotherapy (cisplatin/paclitaxel), or to the direct inhibition of sfRon kinase by BMS777607. Our findings indicate that ovarian tumors expressing sfRon are most effectively treated with multi-kinase inhibitors simultaneously targeting AKT and S6K1, such as AD80, which results in long-term anti-tumor response and prevents metastasis development.

## INTRODUCTION

High-grade serous ovarian cancer (HG-SOC) is the most common and aggressive subtype of epithelial ovarian cancer [1]. Since the 1970’s, standard treatment has been debulking surgery followed by platinum–taxane chemotherapy. Unfortunately, this treatment rarely results in a durable response, eventually leading to recurrence of chemotherapy-resistant tumor in ~85% of patients and the five-year survival rate of only 30% [2-4]. Precision medicine holds a great promise for the improvement of ovarian cancer treatment and has already led to FDA approval of individualized therapies including the anti-angiogenic agent bevacizumab and poly(ADP-ribose) polymerase (PARP) inhibitors [5, 6].

In this study, we explored a novel precision medicine approach in ovarian cancer by validating a multi-kinase inhibitor strategy targeting short-form Ron (sfRon) pathway to achieve a sustained anti-tumor response. This work stems from our previous studies demonstrating that the N-terminally truncated isoform of Ron receptor known as sfRon drives ovarian cancer progression [7]. The constitutively active sfRon kinase preferentially signals through the PI3K pathway, which is associated with enhanced proliferation and invasion of cancer cells *in vitro* and *in vivo* [7-9]. Our previous studies showed that the sfRon pathway can be successfully targetable in cancers by Ron kinase inhibitor BMS777607 or PI3K inhibitor BKM120, respectively. However, these treatment regimens were unable to achieve long-lasting tumor regression after treatment cessation [9]. Although targeted therapies have revolutionized the practice of oncology, still many cancers outsmart such precision-medicine efforts, which may lead to drug resistance and tumor recurrence [10, 11]. This has important implications for targeting the sfRon pathway, especially since sfRon strongly induces PI3K signaling. The PI3K/AKT/mTOR signaling network is well recognized for its complexity, crosstalk and feedback control, and is one of the most challenging pathways to successfully target [12-14]. Therefore, we hypothesized that the most effective strategy to prevent drug resistance and maximize anti-cancer efficacy is to simultaneously inhibit the key targetable regulators within sfRon pathway to prevent or circumvent a parallel oncogenic signaling that promotes tumor progression [10, 11]. Using a detailed dissection of cellular signaling pathways *in vitro*, we demonstrated that S6K1 is a key component of a multi-kinase targeting strategy in sfRon expressing ovarian tumors. We discovered that the multi-kinase inhibitor AD80 that targets both AKT and S6K1, prominently inhibited sfRon downstream pathway without compensatory reactivation of the mTORC1-S6K1 upstream signaling. Using human ovarian xenografts and clinically relevant patient-derived xenografts (PDXs), we showed here, that *in vivo* treatment with single agent AD80 shows superior efficacy to a standard-care chemotherapy (cisplatin/paclitaxel), or to the direct inhibition of sfRon by BMS777607. Further, our data demonstrated that the AD80 therapy resulted in long-term antitumor response completely blocking metastasis development.

Altogether, our study validates AD80 multi-kinase inhibitor as a promising targeted therapy for sfRon-expressing ovarian tumors.

## RESULTS

### The sfRon is not expressed in healthy human fallopian tubes but becomes overexpressed in 50% of patient-derived high-grade serous ovarian cancer (HG-SOC) models

**Figure 1:**
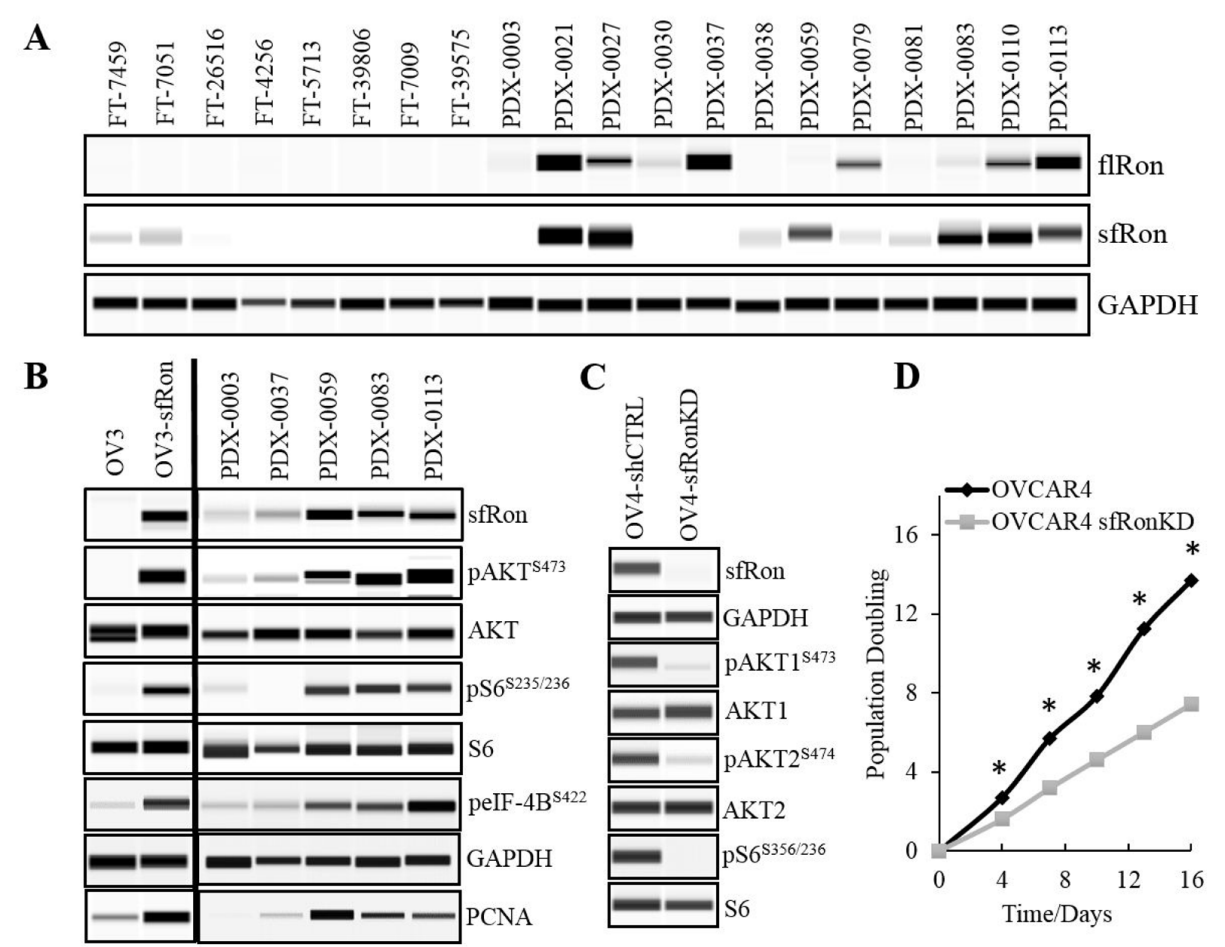
Evaluation of the Ron receptor isoforms expression and the activity of sfRon signaling pathways in ovarian tumors. **A**. WES analysis of fallopian tubes “FT” or patient-derived xenografts (PDX) assayed for flRon and sfRon. Ron isoforms show independent expression pattern in established ovarian PDXs (Fisher’s exact test P = 0.5671). **B**. The sfRon downstream pathway assessed by WES in ovarian cancer cell lines (OVCAR3 cells are denoted as “OV3”) and PDXs that express or lack sfRon expression. **C**. The shRNA-mediated knockdown (KD) of sfRon in OVCAR4 cell line (OV4) leads to suppression of sfRon downstream signaling reflected as loss of phosphorylation of both AKT isoforms (AKT1 and AKT2) and ribosomal protein S6 as compared with control cells expressing construct containing shRNA with a scrambled sequence (shCTRL). **D**. The graph represents the effect of sfRon KD on OVCAR4 cell proliferation assessed by 3T5 cell doubling assay. Each point on the curve is an average measurement of cell count from a three plates followed over the course of the experiment. Asterisks indicate statistically significant difference (P < 0.05) in cell population doubling time between cell lines (unpaired t test).

Since the focus of our research is on HG-SOC subtype that originates from fallopian tubes (15), here we analyzed and compared the full-length Ron (flRon) and short-form Ron (sfRon) levels in human fallopian tubes and PDXs derived directly from patient’s HG-SOC tumors. WES analysis revealed that both Ron receptor isoforms are not expressed in fallopian tubes. In contrast, the protein expression of flRon and sfRon was observed in 50% of HG-SOC PDXs (Figure 1A). With the number of tissues examined here, we report that the flRon and sfRon isoforms become independently activated and expressed in established ovarian PDXs (Fisher’s exact test, P = 0.0123), which is in agreement with published studies [7, 8]. Previous findings demonstrated that the transcription of full-length and short-form Ron is initiated by the two independent promoters within RON (MST1R) gene, each regulated by distinct epigenetic mechanisms that leads to an independent expression of Ron isoforms in malignant tumors [8, 16-18]. Since our previous work shows that the sfRon plays a superior role to flRon in ovarian cancer progression [7], in this paper, we focus specifically on the sfRon isoform and novel strategies to effectively target this receptor.

### The sfRon expression is associated with high activity of the PI3K-AKT-mTORC1-S6K1-S6 signaling axis and increased proliferation of cancer cells 

Our previous mechanistic studies revealed that sfRon preferentially signals through the PI3K pathway, which promotes tumor growth [7, 8]. Here, we performed a detailed analysis of sfRon downstream signaling in ovarian cancer cell lines and PDXs by WES. Consistent with our previous studies, we observed that sfRon expression is associated with high activity of PI3K pathway. Further analysis revealed that sfRon induces AKT-mTORC1-S6K1-S6 signaling, including S6K1 downstream targets such as ribosomal protein S6 and eukaryotic initiation factor 4B (eIF-4B) (Figure 1B). Previous reports show that S6K1 is a hub for translation control that regulates protein synthesis in dividing cells and enhances cell proliferation [19, 20]. In agreement with these findings, we found that sfRon expressing cell lines and PDXs exhibited increased levels of proliferation marker PCNA (Figure 1B). Further studies showed that the shRNA-mediated sfRon knockdown (KD) in OVCAR4 cell line leads to the inhibition of PI3K pathway reflected as loss of the phosphorylation of AKT isoforms (AKT1 and AKT2) and ribosomal protein S6 (Figure 1C). To evaluate if sfRon depletion reduces cell proliferation rate, we measured the cumulative population doublings of cells. Our data showed that OVCAR4-sfRonKD cells proliferate significantly slower (7 population doublings) than the parental OVCAR4 cells (14 population doublings) over the same period of time (Figure 1D).

Together, these data indicate that sfRon activates signaling pathways essential for stimulating anabolic processes such as protein translation and synthesis that may promote robust ovarian tumor growth [21].

**Figure 2:**
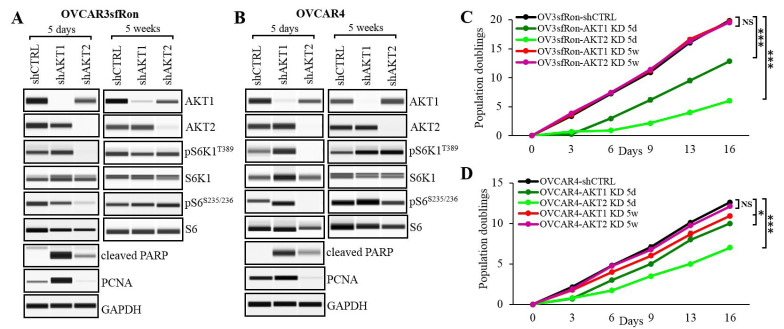
Evaluation of distinct roles of AKT isoforms in ovarian tumors expressing sfRon **A**. and **B**. WES analysis of sfRon signaling network in OVCAR3sfRon and OVCAR4 cell lines genetically depleted from individual AKT isoforms (AKT1 or AKT2) by respective shRNA constructs vs. control cells containing shRNA with a scrambled sequence (shCTRL). The sfRon signaling was evaluated in short (5 days) and long (5 weeks) term culture conditions. **C**. and **D**. The graph represents the effect of individual AKT isoforms KD on OVCAR3sfRon and OVCAR4 cell proliferation. 3T5 cell doubling assay was performed in cell lines 5 days (5d) or 5 weeks (5w) following shRNA-mediated silencing of respective AKT isoform. The following symbols indicate the statistical significance of data: NS = not significant, * = *p* < 0.05 and *** = *p* < 0.001 (multiple t test).

### Distinct roles of AKT1 and AKT2 in regulating ovarian cancer cell proliferation and survival

AKT is a key effector kinase in the PI3K pathway that has been hyperactivated in ovarian tumors expressing sfRon [7]. Since there is a growing evidence that different AKT isoforms have non-redundant functions, we performed experiments to dissect the specific roles of AKT isoforms in ovarian cancer [22]. We focused on AKT1 and AKT2 isoforms, which are expressed in most tissues and have been implicated in ovarian cancer pathogenesis in multiple studies [23-25]. We performed shRNA-mediated knockdown of AKT1 or AKT2 in OVCAR3sfRon and OVCAR4 cell lines, and assessed the effects of each isoform depletion on cell functions. In both cell lines, AKT1 or AKT2 were specifically silenced 5 days following the knockdown (Figure 2A, 2B, left panels). Interestingly, in AKT1 KD cell lines we did not observed the suppression of mTORC1-S6K1-S6 cascade, which is a canonical PI3K/AKT downstream signaling (Figure 2A, 2B, left panels). A cell proliferation assay revealed that AKT1 KD cell lines proliferate significantly faster than AKT2 KD counterparts, but markedly slower than control cells (Figure 2C, 2D). Slower cell proliferation could be a result of an increased apoptosis induced by the loss of AKT1 isoform (reflected as increased levels of apoptosis marker cleaved PARP). In contrast to AKT1 KD, the genetic depletion of AKT2 resulted in complete inhibition of mTORC1-S6K1-S6 signaling (Figure 2A, 2B, left panels). We also observed a loss of proliferation marker PCNA and a prominent inhibition of cell proliferation rate in these cells (Figure 2C, 2D). Moreover, AKT2 KD cells demonstrated significantly lower levels of apoptosis marker cleaved PARP than control or AKT1 KD counterparts.

Together, our findings demonstrated distinct roles of AKT isoforms, where AKT1 regulates cell survival, rather than cell proliferation, while AKT2 regulates cell proliferation through induction of mTORC1-S6K1-S6 signaling.

**Figure 3:**
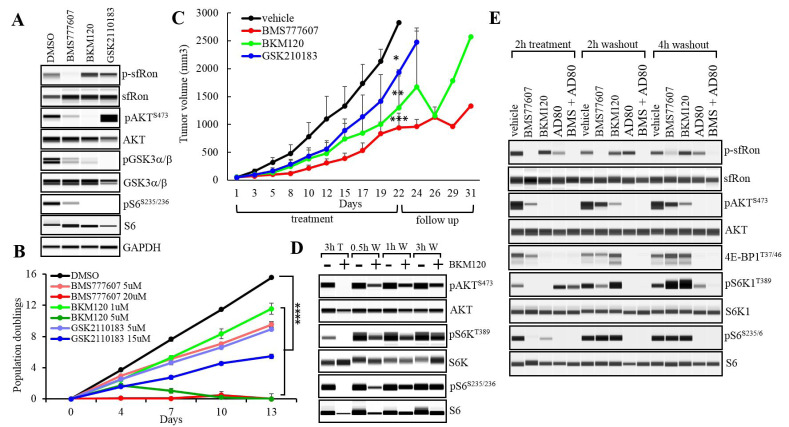
The sfRon pathway inhibition kinetics. **A**. WES analysis of OVCAR3sfRon cell lysates harvested following 2h in vitro treatment with sfRon pathway inhibitors. We noted a feedback increase in AKT phosphorylation following treatment with GSK2110183 as previously reported with other ATP competitive AKT kinase inhibitors. **B**. The effects of sfRon pathway suppression by selective inhibitors on OVCAR3sfRon cell proliferation assessed by 3T5 cell doubling assay (**** = *p* < 0.0001, one way ANOVA). **C**. Graph represents OVCAR3sfRon tumor growth rate in NOD/SCID mice. Animals were treated 5 days per week for 3 weeks with vehicle, BMS777607 (60 mg/kg), BKM120 (60 mg/kg) and GSK2110183 (60 mg/kg). Asterisks indicate statistically significant difference in tumor volumes at the last day (day 22) of drug treatment (* = *p* < 0.05, ** = *p* < 0.01 and *** = *p* < 0.001, one way ANOVA). **D**. Evaluation of the target-specific inhibition kinetics of PI3K inhibitor BKM120. OVCAR3sfRon cells were treated with 10µM of BKM120 for 3h (T), then BKM120 was washed off (W), and the phosphorylation kinetics of downstream targets determined by WES at various time points. E. Evaluation of the target-specific inhibition kinetics of selected sfRon pathway inhibitors. OVCAR3sfRon cells were treated for 2h with BMS777607 (1µM), BKM120 (1µM), AD80 (1µM) and combination of BMS777607 (1µM) and AD80 (1µM), then drugs were washed off, and the phosphorylation kinetics of downstream targets determined by WES at various time points.

### Depletion of AKT isoforms activates a compensatory mechanism that gradually restores the AKT-specific signaling and function

We observed that ovarian cancer cell lines that have been stably depleted from AKT isoforms regained their aggressive tumor phenotype such as enhanced cell proliferation over a period of several weeks. We re-evaluated the expression of specific AKT isoforms in ovarian cancer cell lines that have been cultured for at least 5 weeks and confirmed that the expression of respective AKT isoforms remained completely suppressed (Figure 2A, 2B right panels). Further analysis revealed that despite the complete inhibition of individual AKT isoforms in long-term culture conditions, the AKT-specific downstream signaling and tumor promoting functions have been restored. For instance, the AKT2-specific mTORC1-S6K1-S6 signaling has been restored to the levels found in control cells resulting in increased cell proliferation (Figure 2A, 2B right panels, 2C and 2D). In summary, we show that the ablation of either AKT isoform leads to the induction of compensatory mechanisms restoring the AKT-specific function within sfRon pathway.

### Inhibition of sfRon pathway by selective small molecule inhibitors significantly reduces proliferation of ovarian cancer cells *in vitro*

We showed here, that the depletion of AKT isoforms, eventually leads to the activation of compensatory mechanisms restoring AKT-specific signaling and cell proliferation. These results prompted us to evaluate the effects of pharmacological inhibition of different components of sfRon pathway to assess the anti-cancer efficacy and potential compensatory responses of each treatment. We used several small molecule compounds such as Ron kinase inhibitor BMS777607 [26], PI3 kinase inhibitor BKM120 [27] and pan-AKT kinase inhibitor GSK2110183 [28]. We tested the ability of these compounds to specifically inhibit their targets *in vitro* using OVCAR3sfRon cell line. We used drug doses optimized by our previous studies or recommended by manufacturer [9]. Within 2h of the treatment with BMS777607 (5µM), BKM120 (1µM) and GSK2110183 (5µM), all compounds inhibited the phosphorylation of their respective targets such as sfRon, AKT and GSK3α/β (Figure 3A). We observed a feedback increase in AKT phosphorylation following treatment with GSK2110183 as previously reported with other ATP competitive AKT kinase inhibitors (Figure 3A) [29, 30]. Further, we evaluated whether pharmacological inhibition of different components of sfRon pathway reduces cell proliferation *in vitro*. The OVCAR3sfRon cells were grown in 2 different doses of each drug for 2 weeks. We used a minimal drug dose, which was optimized in our lab to have inhibitory effect on drug-specific target, in parallel with a 3-5 fold higher dose (maximal dose). The results revealed that the minimal dose of each drug significantly reduced cell population doublings, while the maximal dose induced stronger suppression of cell proliferation as expected. The most potent cell proliferation inhibition, however, was observed with BMS777607 and BKM120 maximal doses (Figure 3B). Together, these data revealed that the inhibition of sfRon or PI3 kinase exerted more potent anti-proliferative effects on ovarian cancer cells than the inhibition of AKT.

### Inhibition of the sfRon downstream pathway significantly reduces ovarian tumor growth *in vivo*

To test whether sfRon pathway inhibitors could effectively block ovarian cancer growth *in vivo*, we performed drug treatment of NOD/SCID mice carrying subcutaneous OVCAR3sfRon tumors. The treatment included BMS777607 (60 mg/kg), BKM120 (60 mg/kg), GSK2110183 (60 mg/kg) or vehicle (70% PEG in PBS). Drugs were administered by oral gavage 5 times a week for 3 weeks followed by 10-day follow up period. We found that, although all compounds exhibited significant tumor growth inhibition in comparison with control group, the most potent anti-tumor effect was achieved with BMS777607. However, we also observed that after treatment cessation, the tumors accelerated their growth in a follow up period (Figure 3C). 

In our further studies, we aimed to maximize the anti-cancer efficacy by simultaneously blocking several critical regulators within sfRon pathway, which stems from the concept of multi-target therapy [31, 32].

### The sfRon pathway inhibition kinetics

Our *in vivo* data revealed that the treatment targeting individual components of sfRon pathway has a limited long-term efficacy (Figure 3C). To get better insight into the potential mechanism associated with tumor progression during treatment or shortly after therapy cessation, we investigated the sfRon signaling inhibition kinetics. Since PI3K cascade is a major pathway activated by the sfRon, which is also known for the complexity of regulation, we selected PI3K inhibitor (BKM120) to perform a pilot study assessing the kinetics of a target-specific efficacy *in vitro*. We treated OVCAR3sfRon cells with 10µM of BKM120 for 3h, then BKM120 was washed off from cells, and the cells were incubated in fresh medium for additional 0.5h, 1h or 3h. WES analysis revealed that during 3h of treatment, the cells demonstrated complete inhibition of PI3K downstream targets: pAKT, pS6K1 and pS6. However, 0.5h after drug removal from cells, the PI3K cascade has been restored (Figure 3D). This study indicate, that ovarian cancer cells are able to rapidly restore PI3K signaling when the PI3K-selective inhibitor is cleared from the system, which might contribute to the recovery of cell proliferation thereby augmenting tumor growth. These findings are in agreement with previous data showing that the maximum inhibition of pAKT by BKM120 is achieved within the first hour, which is then followed by the progressive re-expression of pAKT reaching full AKT activity within 24h [27].

### Treatment with multi-kinase inhibitor AD80 alone or in combination with BMS777607 results in sustained suppression of sfRon pathway

Our data showing the rapid rebound of PI3K signaling shortly after removal of PI3K inhibitor from cancer cells (Figure 3D), promoted us to identify new strategies to reach more sustained inhibition of sfRon downstream pathway. Through literature search, we identified AD80, which is a multi-kinase inhibitor of AKT and S6K1 kinases. To determine if AD80 is able to induce and maintain sustained suppression of sfRon signaling, we compared side-by-side the efficacy of AD80 with other inhibitors of sfRon pathway. OVCAR3sfRon cells were treated for 2h with 1µM dose of BMS777607, BKM120, AD80 and AD80+BMS77607 followed by drug washout and cell culture for 4 more hours in standard medium. The results revealed that all drugs inhibited their respective targets during 2h of treatment (Figure 3E). We also observed that even though AD80 completely suppressed the phosphorylation of S6K1 target S6, the S6K1 remained phosphorylated at T389 site, which could indicate an increase in mTORC1 activity. Further studies showed that the S6K1 phosphorylation was not accompanied by the increase in phosphorylation of the mTORC1 substrate 4E-BP1 (Figure 3E). The S6K1 T389 phosphorylation without concomitant activation of mTORC1 downstream targets has been previously describe with other S6K1 inhibitors [20]. However, after drug(s) removal from cells (washout), the cells treated with BMS777607 or BKM120 resumed their signaling to certain extend, while cells treated with AD80 or AD80+BMS777607 had their target-specific signaling completely suppressed (Figure 3E and Supplementary Table 1). Moreover, our data show that the BMS777607 or BKM120 agents, during 4h drug washout period exhibited robust induction of the mTORC1-S6K1-S6 cascade, while the AD80 or AD80+BMS777607 treatment regimen kept this pathway completely suppressed during washout conditions (Figure 3E and Supplementary Table 1). Next, we validated our findings with other ovarian cancer cell lines expressing sfRon (OVCAR4 and OVSAHO). We obtained similar results demonstrating the more potent inhibition of sfRon downstream signaling during treatment and washout period with AD80 or AD80+BMS777607 regimens vs. single agent BMS777067 (Supplementary Figure 1).

### BMS777607 and AD80 specifically target sfRon pathway

**Figure 4:**
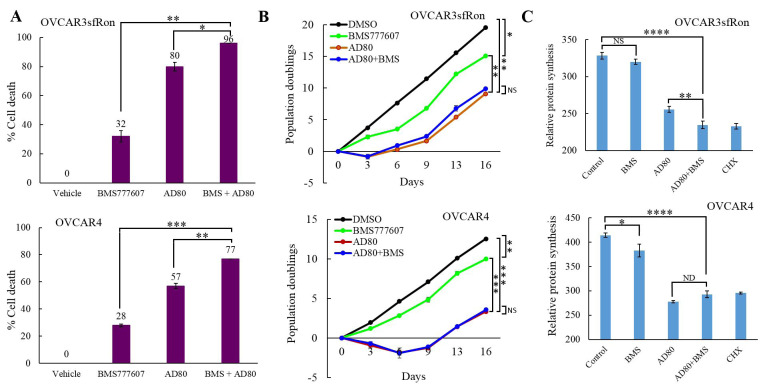
The effects of AD80 and/or BMS777607 treatment on cell proliferation, cell viability and inhibition of protein synthesis. **A**. Graphs represent the results of MTT assay performed on OVCAR3sfRon and OVCAR4 cells. The results show percentage increase in dead cells of drug-treated cells vs. control cells (vehicle) that were assumed to be 100% viable (0% of dead cells). **B**. The effects of 24h pre-treatment of cells with 10µM dose of AD80 and/or BMS777607 agents on cell proliferation (3T5 cell doubling assay). **C**. The effects of 16h pre-treatment of cells with 10µM dose of AD80 and/or BMS777607 agents on protein synthesis inhibition compared with cycloheximide (CHX) treatment serving as positive control. The statistical significance in A., B. and C. was established by using unpaired t test. The following symbols indicate the statistical significance of data: NS = not significant, * = *p* < 0.05, ** = *p* < 0.01, *** = *p* < 0.001 and **** = *p* < 0.0001.

It has been shown that in addition to effectively blocking sfRon/Ron kinase activity [9], the BMS777607 has some inhibitory activity towards other Met family members like Met or TAM receptor tyrosine kinases such as Axl [26]. Similarly, the multi-kinase AD80 compound is a potent inhibitor of AKT and S6K1 kinases as well as other targets including RET and Axl [20, 33]. This prompted us to test if ovarian cancer cell lines and ovarian PDX model (PDX-0113) used in this study express those additional targets, which inhibition could lead to sfRon-independent antitumor efficacy. WES analysis revealed that ovarian cancer cell lines and PDX-0113 lack the expression of Met and Ret. We observed, however, that OVCAR4 cell line expresses Axl, which is a predicted target of both compounds (Supplementary Figure 2). To determine whether BMS777607 and AD80 have off-target effects or whether the expression of Axl in OVCAR4 cell line contributes to an antitumor efficacy of those drugs, we performed proliferation assay and drug response assay. First, we tested whether BMS777607 and AD80 have any inhibitory effects on ovarian cancer cell proliferation in absence of sfRon receptor. Proliferation assay revealed similar results for the two sfRon-negative cell lines (OVCAR3 and OVCAR4-RonKD), where 24h pre-treatment with indicated sfRon pathway inhibitors marginally inhibited cell proliferation without reaching statistical significance (Supplementary Figure 3A). Next, we evaluated the cytotoxic effect of BMS777607, AD80 and AD80+BMS777607 regimens on viability of OVCAR3 and OVCAR4-RonKD cell lines. Our results demonstrated that the BMS777607 and/or AD80 treatment is significantly less effective in killing tumor cells in ovarian cancer cell lines lacking sfRon expression (Supplementary Figure 3B) than their sfRon expressing counterparts (OVCAR3sfRon and OVCAR4 shown in Figure 4A)

Overall, our data demonstrated that, as expected, either BMS777607 or AD80 is not effective in cancer cell lines lacking the sfRon expression. Moreover, the expression of Axl in OVCAR4-RonKD cell line has not contributed to antitumor response reflected as a suppression of cell proliferation or increased cell death following the BMS777607 and/or AD80 treatment (Supplementary Figure 3).

### The combination of AD80 and BMS777607 kills significantly more ovarian cancer cells than each drug alone

In this study, we demonstrated that the AD80 alone or in combination with BMS777607 has a superior efficacy over the other sfRon pathway inhibitors tested here, resulting in a potent and sustained inhibition of sfRon downstream signaling. Next, we tested whether the sustained sfRon pathway inhibition results in increased cancer cell death. Here, we evaluated the cytotoxic effect of BMS777607, AD80 and AD80+BMS777607 regimens on viability of ovarian cancer cells (OVCAR3sfRon and OVCAR4) by drug response assay. The cells were treated for 4 days with 10 µM dose of each drug followed by the assessment of percentage of dead cells. Our data revealed that the combination of AD80 and BMS777607 was the most cytotoxic treatment killing 96% of OVCAR3sfRon cells and 77% of OVCAR4 cells (Figure 4A). Treatment of cells with single agents was less cytotoxic, and showed that the AD80 is a more potent drug in killing cancer cells (80%-57% of dead cells) than the BMS777607 (32%-28% of dead cells) (Figure 4A).

Here, we also evaluated the cytotoxic effect of BMS777607, AD80 and AD80+BMS777607 regimens on viability of OVCAR3sfRon and OVCAR4 cell lines that have been depleted from individual AKT isoforms downstream from sfRon. Our data revealed that cell lines depleted from either AKT1 or AKT2 showed various levels of response to BMS777607 and/or AD80 treatment (Supplementary Figure 4). Our results also demonstrated that the BMS777607 and/or AD80 treatment is less cytotoxic in AKT deficient cell lines (Supplementary Figure 4A, B, D and E) than in their AKT expressing counterparts (Figure 4A). These data indicate that a loss of AKT functions could be associated with a development of resistance to the sfRon pathway targeted therapy.

### AD80 alone or combination with BMS777607 more effectively reduces ovarian cancer cell proliferation than BMS777607 monotherapy

Here, we tested if pharmacological inhibition of sfRon signaling reduces cell proliferation. We treated OVCAR3sfRon and OVCAR4 cell lines with 10µM dose of each drug (AD80, BMS777607 and AD80+BMS777607) for 24h, removed drug(s) from culture medium and measured cell proliferation rate. By using this approach, we aimed to recapitulate the drug pharmacokinetics analogous to that observed in *in vivo* biological systems, where the anti-cancer drugs are being naturally cleared from the body (usually within 24h-48h after administration) [34, 35]. Our data revealed similar results for both cell lines, where 24h pre-treatment with BMS777607 modestly inhibited cell proliferation, while AD80 or AD80+BMS777607 regimens resulted in a significantly stronger reduction of cell proliferation rate (Figure 4B). We also noted that the AD80+BMS777607 treatment did not confer additional benefit in reducing cell proliferation over the treatment with single agent AD80.

### AD80 alone or combination with BMS777607 strongly inhibits protein synthesis in ovarian cancer cells

In this study, we demonstrated that the treatment with AD80 with or without addition of BMS777607 results in a potent and sustained inhibition of mTORC1-S6K1-S6 signaling cascade. Since mTORC1-S6K1-S6 signaling axis is implicated in regulation of protein translation and synthesis, we tested if pharmaceutical inhibition of this pathway by AD80 and/or BMS777607 reduces protein synthesis in ovarian cancer cell lines. To measure protein synthesis inhibition, OVCAR3sfRon and OVCAR4 cell lines were treated with 10µM dose of each drug (AD80, BMS777607 and AD80+BMS777607) for 16h or 180µM dose of protein synthesis inhibitor cycloheximide for 30 min. (positive control). Protein synthesis assay revealed that the treatment with BMS777607 had a minimal effect on protein synthesis inhibition. In contrast, treatment with AD80 or AD80+BMS777607 significantly blocked protein synthesis to similar levels as cycloheximide (Figure 4C). These findings demonstrated that the potent suppression of protein synthesis by AD80 could be a mechanism that impairs tumor growth and/or cancer cell survival.

### The *in vivo* treatment with AD80 alone or combination with BMS777607 results in a potent ovarian tumor growth inhibition 

**Figure 5:**
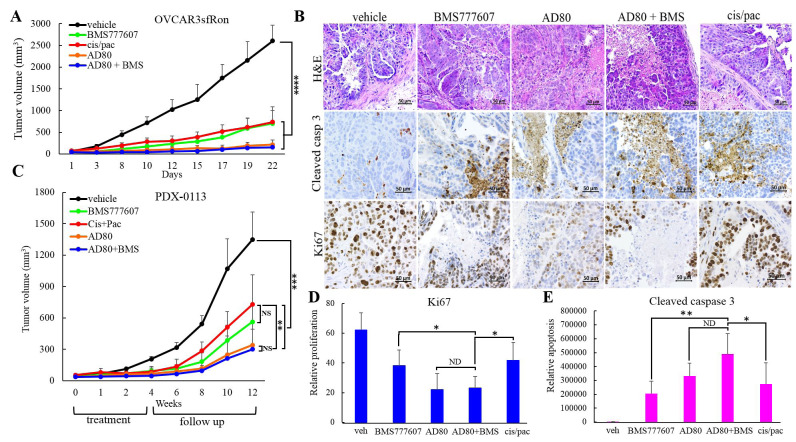
Evaluation the efficacy of AD80 and/or BMS777607 compounds vs. chemotherapy in ovarian tumor models expressing sfRon. **A**. Graph represents OVCAR3sfRon tumor growth rate in NOD/SCID mice. Animals were treated for 3 weeks with vehicle, BMS777607 (60 mg/kg, 4x/wk), AD80 (25 mg/kg, 4x/wk), BMS777607 + AD80 (60 mg/kg, 2x/wk + 25mg/kg, 4x/wk) or cisplatin + paclitaxel (3 mg/kg 1x/wk + 10 mg/kg1x/wk) (one way ANOVA). **B**. Representative sections of tumors obtained from therapeutic experiments on PDX-0113 treated with different drug regimens. Tumor sections were H&E stained and immunohistochemically evaluated for Ki67 or cleaved caspase-3 expression. **C**. Graph represents PDX-0113 tumor growth rate in NOD/SCID mice. Animals were treated for 4 weeks with vehicle, BMS777607 (60 mg/kg, 4x/wk), AD80 (25 mg/kg, 4x/wk), BMS777607 + AD80 (60 mg/kg, 2x/wk + 25mg/kg, 4x/wk) or cisplatin + paclitaxel (3 mg/kg 1x/wk + 10 mg/kg1x/wk), and followed for additional 8 weeks for recurrence to investigate the long-term effect of the treatment (one way ANOVA). **D**. and **E**. Quantification of Ki67 or cleaved caspase-3 positive cells determined from IHC staining of PDX-0113 tumors shown in B. Statistical significance was evaluated using unpaired t test. A-E. The following symbols indicate as follow: x/wk - times per week, NS = not significant, * = *p* < 0.05, ** = *p* < 0.01, *** = *p* < 0.001 and **** = *p* < 0.0001.

To test whether there is an additional benefit of the combination therapy AD80+BMS777607 over AD80 monotherapy *in vivo*, we used OVCAR3sfRon ovarian xenograft model. Therapy consisted of vehicle, BMS777607 (60mg/kg) and AD80 (25mg/kg) administered 4 times a week for 3 weeks. Additional treatment groups included BMS777607+AD80 (BMS777607 60mg/kg given 2 times per week; AD80 25mg/kg given 4 times a week) and IV injections of cisplatin 3mg/kg and paclitaxel 10mg/kg each administered once a week for 3 weeks. All treatment regimens significantly inhibited tumor growth when compared to control group. Treatment with a single agent BMS777607 showed similar efficacy as the efficacy of chemotherapy (cisplatin/paclitaxel). In contrast, AD80 alone, or the combination of AD80+BMS777607 showed a superior efficacy to cisplatin/paclitaxel treatment (Figure 5A).

### Treatment with AD80 alone or combination with BMS777607 results in a sustained ovarian PDX growth inhibition

We extended our *in vivo* studies to patient-derived xenografts (PDXs) to validate short- and long-term effects of AD80 and/or BMS777607 therapy. Our ovarian PDX lines are clinically relevant tumor models that were generated directly from ovarian tumors from patients followed by thorough characterization and validation (Supplementary Figure 5). We selected the PDX-0113 that was confirmed for sfRon expression and absence of Met, Axl and Ret expression (Figure 1A and Supplementary Figure 2). Next, viable PDX-0113 tumor chunks were implanted subcutaneously into left flank of NOD/SCID mice. Treatment regimens and drug doses for *in vivo* PDX-0113 study were the same as described for OVCAR3sfRon ovarian xenograft.

During 4-week treatment period, all treatments significantly inhibited tumor growth when compared to control group. To assess a long-term efficacy of each treatment modality, we monitored the residual tumors for 8 weeks following discontinuation of all treatments. Our data demonstrated that all animal groups showed tumor recurrence at varying time points, where cisplatin/paclitaxel group showed the fastest tumor recurrence. The most effective treatment regimens, however, were both AD80 and AD80+BMS777607, which resulted in the strongest tumor growth reduction and no cancer progression for several weeks after therapy cessation. Eventually, AD80+/-BMS777607 treated tumors started to regrow slowly, reaching significantly smaller tumor volumes compared to chemotherapy treated tumors at the endpoint of experiment (Figure 5C).

To get insight into the effects of different treatments on ovarian tumor morphology and markers of proliferation and apoptosis, we performed H&E and IHC analysis of representative PDX-0113 tumors tissues with the following antibodies: human cytokeratin (CK), PAX8, pS6, Ki67 and cleaved caspase-3 (Figure 5B and Supplementary Figure 6). As shown in Figure 5B, 5D, there is a good correlation between the expression of proliferation marker Ki67 and tumor growth *in vivo* (Figure 5C). The Ki67 staining decreased markedly upon AD80 or AD80+BMS777607 treatment in comparison with vehicle, cisplatin/paclitaxel or BMS777607-treated tumors. Assessment of apoptosis associated with treatment revealed that vehicle-treated PDX-0113 tumors exhibited marginal levels of the apoptotic marker cleaved caspase-3. In contrast, treatment with AD80 and/or BMS777607 and cisplatin/paclitaxel drugs resulted in various levels of apoptosis where AD80 +/- BMS777607 exhibited the strongest apoptotic effect (Figure 5B, 5E).

### Treatment with AD80 and/or BMS777607 inhibits tumor progression and metastasis of advanced orthotopic ovarian tumor model

**Figure 6:**
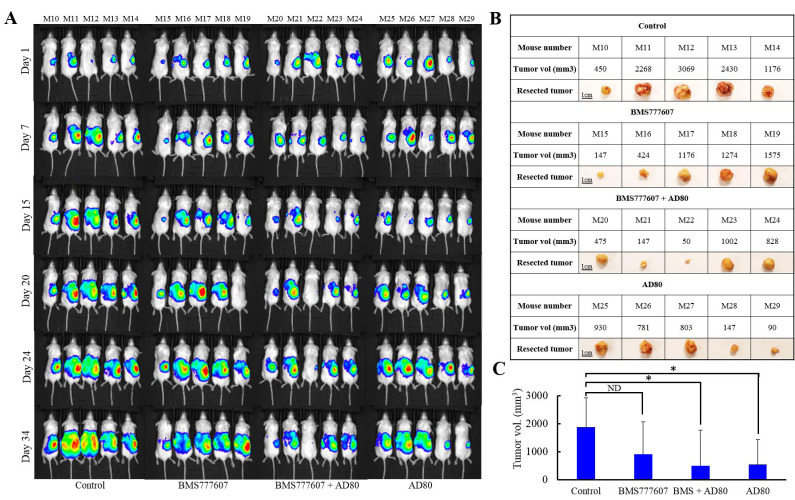
Evaluation of the efficacy of AD80 and/or BMS777607 compounds vs. chemotherapy in advanced orthotopic ovarian tumor model. **A**. Bioluminescence imaging of luciferase activity in NOD/SCID mice orthtopically injected with luciferized OVCAR4 cells. Six weeks after tumor cells inoculation (day 1), animals were treated for 3 weeks with vehicle, BMS777607 (60 mg/kg, 4x/wk), AD80 (25 mg/kg, 4x/wk), BMS777607 + AD80 (60 mg/kg, 2x/wk + 25mg/kg, 4x/wk) or cisplatin + paclitaxel (3 mg/kg 1x/wk + 10 mg/kg1x/wk), and then monitored for additional 2 weeks to assess the effects of each treatment on tumor burden and metastasis. **B**. Table shows image and volume of tumors resected from mice shown in A. **C**. Graph represents average tumor volume of each treatment group (NS = not significant, * = *p* < 0.05, unpaired t test).

To test the efficacy of sfRon pathway inhibitors in a tumor model that better recapitulate the natural ovarian cancer microenvironment, we implanted luciferized OVCAR4 cells directly into a NOD/SCID mouse ovary. This orthotopic ovarian tumor model has been shown to recapitulate the metastatic dissemination pattern characteristic for ovarian cancer disease (36). Orthotopic tumors progression was monitored weekly by IVIS bioluminescence imaging, which is a surrogate measure of tumor burden (Figure 6A) [37]. We allowed tumors to grow to a larger size before treatment to stimulate a more realistic clinical scenario of advanced ovarian cancer. Six weeks after tumor cells inoculation mice began treatment with vehicle, BMS777607 (60mg/kg) and/or AD80 (25mg/kg) administered 4 times a week for 3 weeks. We monitored mice for additional 2 weeks after treatment to assess the effects of each treatment on tumor burden and metastasis development. At the endpoint, we performed necropsy and gross visual examination of abdominal cavity for presence of metastatic lesions. Next, we resected tumors and measured their volumes (Figure 6B, 6C). We observed that AD80 or AD80+BMS777607 treatments significantly reduced tumor progression when compared to vehicle-treated mice. BMS777607 monotherapy showed a tendency to reduce tumor progression, when compared with control, though statistical significance was not observed (Figure 6C). Next, we macroscopically evaluated tumor progression and observed that only control mice developed metastasis. At necropsy, 80% of the control mice showed multiple metastatic lesions in the peritoneal cavity, while mice treated with targeted therapies had only primary tumors without detectable metastases (Supplementary Figure 7).

## DISCUSSION

Precision medicine is an emerging approach for ovarian cancer treatment. However insufficient and/or short-term efficacy of targeted therapies resulting in drug resistance is a widely recognized problem in oncology, and there is still considerable room for improvements [11, 38, 39]. Resistance to targeted therapies is associated with the activation of signaling pathway(s) in a parallel or downstream fashion that bypasses the requirements for a specific oncogenic activity, or due to the induction of negative feedback loop(s) [11]. To address these challenges, we explored multi-kinase inhibition of sfRon pathway as a strategy to prevent drug resistance and achieve a sustained anti-tumor response. Here, we showed that sfRon induces PI3K-AKT-S6K1-S6 signaling axis that positively regulates protein synthesis and cell proliferation. To identify targetable regulators with sfRon pathway, we first investigated the specific roles of AKT isoforms. In general, our data show that AKT1 promotes cell survival, while AKT2 increases cell proliferation through induction of mTORC1-S6K1-S6 signaling (Figure 2). We demonstrated that the depletion of ovarian cancer cell lines from pro-survival AKT1 isoform results in more increased cell death than the depletion of AKT2 isoform (Supplementary Figure 4C, F). However, AKT1 KD cell lines are less sensitive to cell death induced by the sfRon pathway inhibitors, which, in part, could be explained by the loss of druggable target (AKT1) leading to development of resistance to the sfRon targeted therapy (Supplementary Figure 4A, B, D and E). An analysis of the effects of individual AKT isoforms knockdown in ovarian cancer cell lines indicate that the growth suppression (Figure 2C, 2D) shown in the AKT2 KD cell lines could be attributed to the partial inhibition of AKT1 isoform by the AKT2 targeting siRNA (Figure 2A, 2B). It is also possible that AKT1 and AKT2 isoforms may have some overlapping functions. Our findings are in agreement with those reported by Khabele et al. and Noske et al., who concluded that among AKT isoforms, the AKT2 is the most important contributor to ovarian cancer cell proliferation [23, 24]. Conversely, Linnerth-Petrik et al. using murine ID8 ovarian cancer model *in vivo*, reported that the ablation of AKT1 reduces tumor growth, while the ablation of AKT2 has the opposite effect accelerating tumor growth [40]. These contradictory observations could be explained, in part, by the complexity of AKT signaling involving more than 100 non-redundant AKT substrates that are often differently expressed across different cancer lineages [41, 42]. Due to the possibility of opposite biological consequences following the inhibition of the same AKT isoform in different tumors [23, 24, 40], potential overlapping functions, or the activation of unfavorable compensatory mechanisms restoring the AKT function, targeting individual AKT isoforms in cancer may carry an unpredictable clinical outcome.

Recently, there has been an encouraging shift towards development of multi-target treatment strategies to improve cancer therapy [31-33, 43, 44]. This prompted us to undertake an approach that relays on targeting several kinases within sfRon pathway (vertical inhibition) to prevent induction of compensatory mechanisms and drug resistance [31, 32]. We compared the efficacy of single- vs. multi-kinase inhibition of sfRon pathway. Our *in vitro* studies revealed that a multi-kinase inhibitor AD80 is the most potent single agent that completely suppressed the sfRon pathway, even hours after drug has been washout from cells. In contrast, treatment with selective sfRon or PI3K kinase inhibitors led to signaling restoration shortly after treatment cessation (Figure 3E). Our findings are in agreement with Liu et al. showing a durable suppression of S6K1-induced signaling by AD80 in PTEN-deficient tumors [20]. Similarly, Yu et al., identified an AD80 as a lead compound profoundly inhibiting oncogenic signaling in hepatocellular carcinoma [45].

In this study, we also showed that the inhibition of ribosomal protein S6 is a good indicator of a protein synthesis suppression, which could be a mechanism that impairs ovarian tumor growth (Figure 3E and Figure 4C). Accordingly, the sustained inhibition of S6 by AD80 resulted in a potent suppression of cell proliferation and tumor growth, while treatment with BMS777607 had only a minimal inhibitory effect on protein synthesis, resulting in modest tumor growth inhibition (Figure 4C and Figure 5A, 5C). Together, our findings support the notion that an increased protein synthesis requirement for rapidly proliferating cancer cells could be a druggable vulnerability of malignant tumors and a potential new strategy to target sfRon expressing ovarian tumors [46, 47].

We further validated the promising multi-kinase treatment strategy using clinically-faithful PDX-0113 and advanced orthotopic xenograft (OVCAR4). The PDX study demonstrated that the benefit of targeted therapies over chemotherapy was not apparent until treatment stopped (likely due to the slow intrinsic growth rate of PDX-0113). However, during the 8-week follow up period the AD80 or AD80+BMS777607 regimens resulted in the most durable responses superior to chemotherapy (Figure 5C). To overcome the shortcoming of previous tumor models in recapitulating the natural ovarian cancer microenvironment, we used orthotopic ovarian cancer xenograft (OVCAR4). In advanced orthotopic ovarian tumor model, we observed significant reduction of tumor progression with AD80 or AD80+BMS777607 treatments, while the single agent BMS77607 suppressed tumor growth without reaching statistical significance (Figure 6). In addition, all targeted therapies prevented metastasis development in contrast to control mice that showed multiple metastatic lesions in the peritoneal cavity (Supplementary Figure 7).

One critical aspect to improve cancer treatment is not only to inhibit the primary oncogenic pathway that reduces cell proliferation, but to simultaneously prevent functional redundancies and pathway crosstalk that facilitate cancer cells survival and drug resistance. We demonstrated for the first time, that targeting sfRon downstream pathway at several fragile nodes (AKT, S6K1) by multi-kinase compound AD80 is the most effective strategy to achieve a sustained tumor growth inhibition and suppression of metastatic disease. There is no additional benefit of combination therapy AD80+BMS777607 over single agent AD80 treatment on tumor growth inhibition. It is possible that comparable antitumor effects of AD80 and AD80+BMS777607 regimens could be a result of inhibiting overlapping targets. For instance, BMS777607 inhibits sfRon kinase resulting in the inhibition of its downstream signaling including AKT and S6K1, while AD80 directly suppresses AKT and S6K1. In addition, Axl and other TAM receptor tyrosine kinases are another potential overlapping targets of BMS777607 and AD80, however in our current (Supplementary Figure 3) and previous studies (9), we have not observed any antitumor efficacy of BMS777607 or AD80 in tumors lacking the sfRon expression while expressing other Met or TAM receptors.

In this study, we tested sfRon targeted therapy in the context of high-grade serous ovarian cancer, which is a prevalent (70% of all ovarian carcinomas) ovarian cancer subtype (48). Our studies show that sfRon is expressed in approximately 50% of HG-SOC tumors, which reinforces the idea that the large proportion of patients diagnosed with ovarian cancer could benefit from a novel therapy targeting the sfRon pathway. It is important to mention, however, that the expression and significance of Ron receptors have not been thoroughly explored in less common non-HG-SOC ovarian cancer subtypes. Thus, the efficacy and applicability of sfRon targeted therapy in those non-HG-SOC malignancies needs rigorous pre-clinical validation before it can be considered for testing in clinical settings.

## MATERIALS AND METHODS

### Source of cells and tumors

OVCAR3 cells expressing sfRon (OVCAR3-sfRon) were previously described [7]. Parental OVCAR3 cell line was purchased from ATCC. OVCAR4 cell line was a gift from dr. Jones [49]. OVSAHO cell line was purchased from JCRB Cell Bank. OVCAR cell lines were authenticated by short tandem repeat (STR) profiling by ATCC. To ensure the identity and validity of our cell lines and to prevent potential problems associated with cell culture, such as cell line misidentification, contamination and genetic drift, we purchase cell lines form validated, reliable source and cryopreserve 20 1 ml vials of each cell line at low passage (passage 1-3). The vials of low passage cell lines are kept protected in lab cell line bank and distributed to lab members according to the experimental needs. All cell lines were maintained in RPMI 1640 Medium containing L-glutamine 300 mg/L (#11875-093, Gibco) supplemented with 10% fetal bovine serum (FBS) (#F0926, Sigma-Aldrich), in a standard humidified incubator at 37ºC in 5% CO2 and 95% O2 atmosphere. Cell lines were tested for Mycoplasma by Idexx BioAnalytics and were found negative for any contamination. PDX-0113 tumor model was obtained from our own PDX collection at the PDX-PCT core facility at OMRF (https://pdx.omrf.org).

### Generation of lentiviruses and cell transduction

To knockdown AKT1, AKT2 or MSTIR gene we used validated shRNA clones (TRCN0000221552, TRCN0000265834 and TRCN0000379811, respectively) from Sigma-Aldrich. Each shRNA was cloned into pLKO.1-puro lentiviral vector (Sigma-Aldrich). Recombinant lentiviruses were produced in HEK293T cells according to standard protocols [9]. Ovarian cancer cells were then infected with lentiviruses containing the respective shRNAs, or control shRNA with a scrambled sequence, followed by selection with puromycin, as described [9].

### Drugs and reagents

BMS777607, BKM120 and AD80 were purchased from Selleckchem as lyophilized compounds. BMS777607 was prepared as a 500 mg/ml stock solution in DMSO. BKM120 was dissolved in 1 volume of NMP (N-methyl-2-pyrrolidone) and 9 volumes of PEG300. AD80 was prepared by adding solvents to the compound individually as follow: 2% DMSO, 30% PEG300, 2% Tween80 and ddH2O. Cisplatin (Alvogen) and paclitaxel (Actavis) were purchased from University of Oklahoma Pharmacy and diluted to desired concentrations in saline or PBS, respectively.

### WES (capillary-based Western blot) and immunoprecipitations

To prepare whole cell lysates, cells or tissues were lysed in Buffer B as described previously [7]. For immunoprecipitations, 200 μg whole cell lysate was diluted in IP buffer and immunoprecipitated with 4G10 anti-phosphotyrosine agarose conjugate (05-777, Millipore) as described previously [9]. Whole cell lysates and immunoprecipitates were processed by WES to separate and visualize cellular proteins according to a standard instrument protocol. Primary antibodies used were: Ron β (#sc-374626, 1:25), GAPDH (#sc-25778, 1:300), PCNA (#sc-56, 1:25) from Santa Cruz Biotechnology; pan AKT (#4691, 1:25), phospho-AKT (#9271, 1:25), S6 (#2217, 1:25), phospho-S6 (#4856, 1:25), phospho-eIF4B (#3591, 1:25), phosphor-AKT1(# 9018, 1:25), phosphor-AKT2 (# 8599, 1:25), AKT1(# 2938, 1:25), AKT2 (# 3063, 1:25), phospho-S6K1 (#9234, 1:25), S6K1 (#2708, 1:25), cleaved PARP (#9541, 1:25), phospho-GSK3α/β (#9331, 1:25), GSK3α/β (#5676, 1:25), Met (#8198, 1:25), Ret (#14556, 1:25) and Axl (#8661, 1:25) from Cell Signaling Technology. Secondary antibodies were included in a Wes Master Kit (PS-MK14, ProteinSimple).

### 3T5 cell proliferation assay

3T5 cell growth assay was performed by plating 5x105 cells per 10 cm tissue culture plate (each cell line was set up in triplicate), followed by counting and re-plating at the same density every 3 days for 13-16 days, as indicated. Population doubling time was calculated using the formula ln(post-3-day cell count/5x105)/ln(2). The given population doubling time was added to the cumulative doubling time of the previous count.

### Dose response assay

Exponentially growing cells were treated *in vitro* with drugs followed by an MTT assay to measure cell viability using the Quick Cell Proliferation Assay kit II (BioVision). Briefly, cells were seeded in a 96-well plate at a density of 25,000 cells/well. After 24h culture, the cells were exposed to desired concentration of drug(s) or vehicle control for 4 days. Next, cells were incubated with the WST reagent for 2h, and absorbance was determined at 450 nm. The results were presented as percentage of dead cells compared with untreated controls. Normalized values were plotted as an average ± SD of three wells per condition.

### Protein synthesis assay

Protein synthesis inhibition was measured using the Protein Synthesis Assay kit (Cayman chemicals). Briefly, cells were seeded in a 96-well plate in a density of 50,000 cells/well. Eight hours after seeding, the cells were exposed to selected drugs for 16h or to cycloheximide for 30 min. (positive control). Next, cells were incubated with O-propargyl-puromycin (OPP), fixed and stained with 5 FAM-Azide for colorimetric detection of OPP-labeled peptides at 450 nm [50].

### Animal experiments

All animal procedures were approved by the OMRF’s Institutional Animal Care and Use Committee. For *in vivo* experiments, 6 week-old female NOD/SCID mice (#1303, Jackson Laboratory) were implanted subcutaneously into the left flank with tumor cells (3x106 OVCAR3sfRon cells) or with PDX-0113 tumor fragment. For orthotopic tumor model, 5x105 luciferized OVCAR4 cells were injected into the mouse ovary as previously described [7]. Mice with established subcutaneous tumors of ~50 mm3 volume were randomized and treated with the indicated drug(s). Mice bearing orthotopic tumors began treatment 6 weeks after tumor inoculation. Subcutaneous tumor volumes were calculated using the formula ½ (Length × Width2). Orthotopic tumors progression was monitored weekly by IVIS bioluminescence imaging (Xenogen IVIS, Xenogen), coupled to the LivingImage analysis software (Xenogen). Before imaging, mice received intraperitoneal (IP) injections of 150 mg/kg luciferin (Gold Biotechnology). Bioluminescence was quantified and expressed as total flux [p/s]. Peritoneal luciferase activity was correlated with the distribution and size of ovarian tumors. At necropsy, orthotopic tumors were harvested and measured with vernier caliper. Mice were monitored weekly for body weight, development and progression of ovarian tumors, and any symptoms of physical distress or illness. At necropsy mice underwent visual inspection of peritoneal tumor load and presence of metastasis.

### Morphologic and immunohistochemical (IHC) analyses of tumors

Two mice bearing PDX-0113 from each group were sacrified 3h after drug(s) administration. Harvested tumors were fixed in 10% neutral buffered formalin, paraffin embedded, and hematoxylin–eosin (H&E) stained according to our standard protocols [7, 9]. Tumors were analyzed by IHC for expression of the following markers: anti-human cytokeratin (1:400, DAKO #Z0622), PAX8 (1:1000, Abcam, #ab189249), WT1 (1:1250, Cell Signaling, #83535), phospho-S6 (1:200, Cell Signaling, #4856), Ki67 (1:200, Thermo Scientific #RM-9106-S1) or cleaved caspase-3 (1:250, Cell Signaling #9661). Staining was visualized by 3,3-diaminobenzidine (DAB), with hematoxylin as a counter-stain. Slides were imaged on an Olympus Bx50 microscope with a Canon EOS Rebel XSI camera using EOS imaging software. For Ki67 or cleaved caspase-3 quantification, 3 images per tumor from two different animals from each treatment group were analyzed with ImageJ software [51]. The relative proliferation (Ki67) or apoptosis (cleaved caspase-3) were expressed as a number of positively stained nuclei in each image, as previously described [52].

### Statistical analysis

All *in vitro* experiments were performed three times and in triplicate when applicable. Values are presented as mean ± SD. Statistical analysis of *in vitro* assays was done using Fisher’s exact test, unpaired t-test or multiple t-test, whenever applicable. The effects of *in vivo* treatment were assessed using analysis of variance (ANOVA) and appropriate post-hoc test. *p* < 0.05 were considered significant. Statistical analysis was performed using GraphPad Prism 6.0 Software.

## SUPPLEMENTARY MATERIALS TABLES AND FIGURES



## Abbreviations

ATCC: American Type Culture Collection
BKM: BKM120
BMS: BMS777607
CHX: cycloheximid
CK: cytokeratin
CTRL: control
DAB: 3,3-diaminobenzidine
DMSO: dimethyl sulfoxide
eIF-4B: eukaryotic initiation factor 4B
FBS: fetal bovine serum
FDA: food and drug administration
flRon: full-length Ron
H: hour
H&E: hematoxylin and eosin
HG-SOC: high-grade serous ovarian cancer
IHC: immunohistochemistry
IV: intravenous
KD: knockdown
Min: minutes
NMP: N-Methyl-2-Pyrrolidone
NS: *not significant *
OMRF: Oklahoma Medical Research Foundation
OPP: O-propargyl-puromycin
OV3: OVCAR3
OV4: OVCAR4
PARP: poly(ADP-ribose) polymerase
PDX: patient-derived xenografts
PEG300: poly(ethylene glycol)300
p: phospho
SD: standard deviation
sfRon: short-form Ron
shRNA: short harpin RNA
STR: short tandem repeat
WES: capillary-based Western blot
wk: week
