# Supplementary material for: Multi-kinase targeted therapy as a promising treatment strategy for ovarian tumors expressing sfRon receptor
Source: Genes Cancer. 2020 Jul 22;11(3-4):106–21. doi: 10.18632/genesandcancer.205 (PMC7805538; doi:10.18632/genesandcancer.205)
Supplement: Supplementary file 1 [file ganc-11-106-s001.pdf]

Multi-kinase targeted therapy as a promising treatment strategy for ovarian tumors expressing sfRon receptor – Wang et al

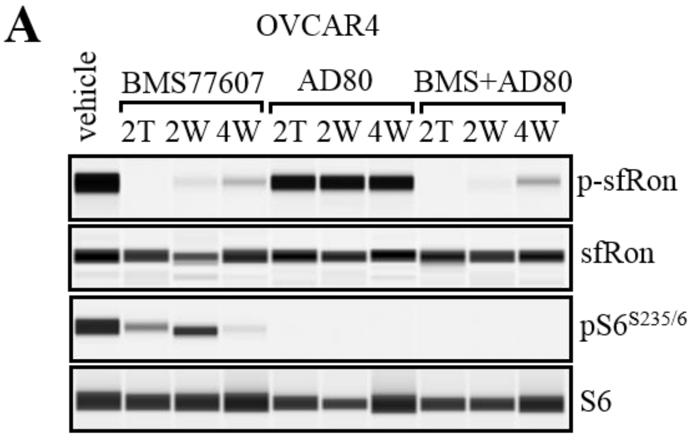

% change of protein expr.	Veh	BMS -2T	BMS – 2W	BMS – 4W	AD80 – 2T	AD80 – 2W	AD80 – 4W	BMS+AD 80 – 2T	BMS+AD 80 – 2W	BMS+AD 80 – 4W
p-sfRon	100	0	15	24	68	62	65	13	14	28
pS6$^{S235/6}$	100	62	72	24	2	1	0	1	0	1

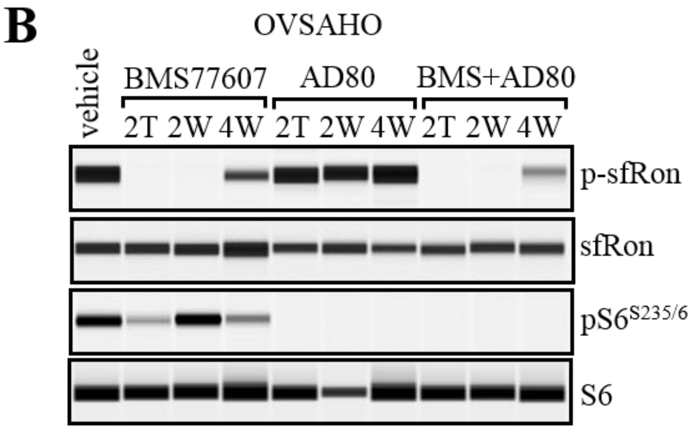

% change of protein expr.	Veh	BMS -2T	BMS – 2W	BMS – 4W	AD80 – 2T	AD80 – 2W	AD80 – 4W	BMS+AD 80 – 2T	BMS+AD 80 – 2W	BMS+AD 80 – 4W
p-sfRon	100	27	22	61	100	92	113	24	25	59
pS6$^{S235/6}$	100	64	113	57	5	3	2	4	3	2

Supplementary Figure 1: Evaluation of a target-specific inhibition kinetics of selected sfRon pathway inhibitors. OVCAR4 cells **A.** or OVSAHO cells **B.** were treated for 2h (2T) with BMS777607 (1 M), AD80 (1 M) and combination of BMS777607 (1 M) and AD80 (1 M). Next, the drugs were washed off, and the cells were incubated for additional 2h (2W) or 4h (4W) followed by the assessment of phosphorylation kinetics of downstream targets by WES. Protein expression was quantified using Compass for Simple Western software and represented as percentage change in the expression of selected phosphorylated proteins in each drug treatment condition, assuming 100% protein expression in control (vehicle-treated) cells.

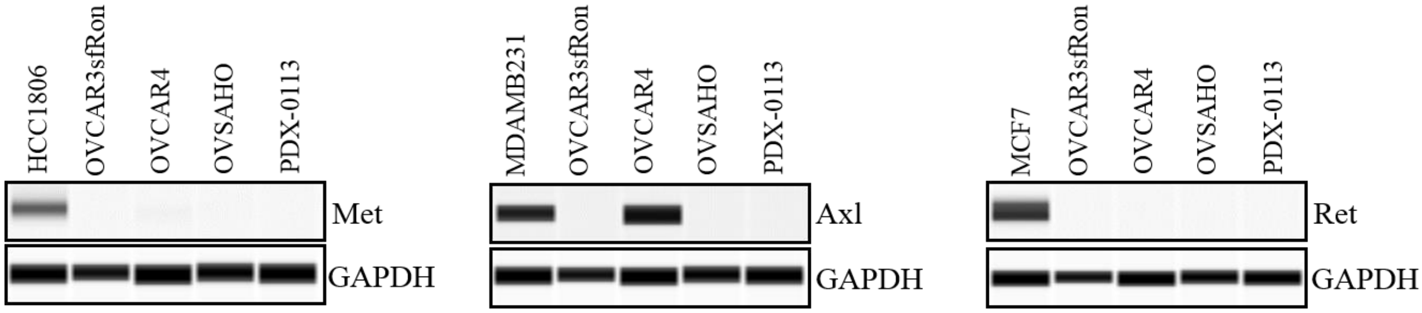

Supplementary Figure 2: Evaluation of the expression of other than sfRon targets of BMS777607 (Met and Axl) and AD80 (Ret and Axl) in ovarian tumor models. WES analysis of ovarian cancer cell lines and ovarian PDX-0113 assayed for Met, Axl and Ret. Breast cancer cell lines that are known to express Met (HCC1806), Axl (MDAMB231) and Ret (MCF7) serve as positive controls. OVCAR4 is the only cell line that expresses predicted target (Axl) of BMS777607 and AD80.

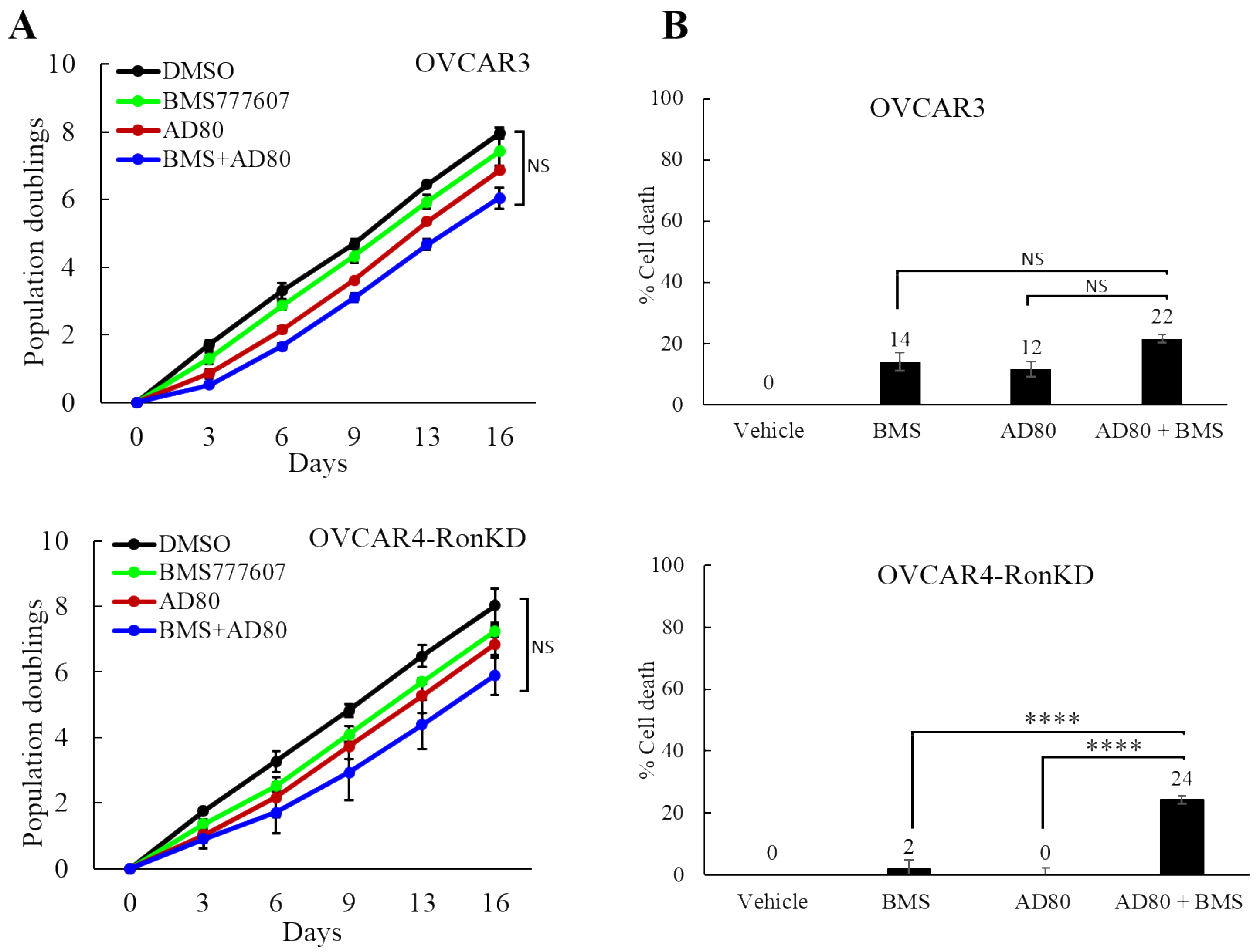

Supplementary Figure 3: The effects of AD80 and/or BMS777607 treatment of ovarian cancer cell lines lacking the sfRon expression on cell proliferation and viability. A. The effects of 24h pre-treatment of cells with 10 M dose of AD80 and/or BMS777607 agents on cell proliferation (3T5 cell doubling assay). **B.** Graphs represent the results of MTT assay performed on OVCAR3 and OVCAR4-RonKD cells. The results show percentage increase in dead cells of drug-treated cells vs. control cells (vehicle) that were assumed to be 100% viable (0% of dead cells). ***A.*** and **B.** was established by using unpaired t test. The following symbols indicate the statistical significance of data: NS = not significant, * = $p < 0.05$, ** = $p < 0.01$, *** = $p < 0.001$ and **** = $p < 0.0001$.

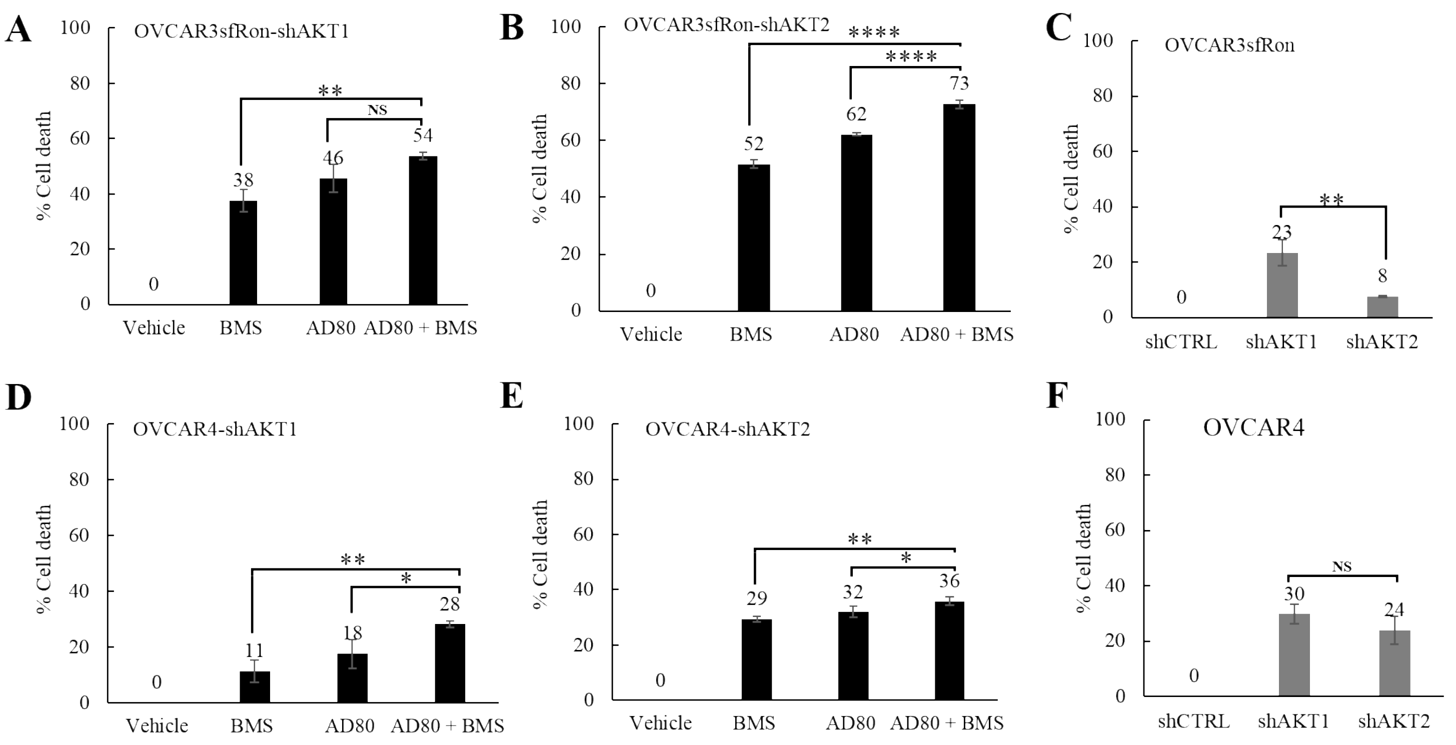

Supplementary Figure 4: The effects of AD80 and/or BMS777607 treatment of ovarian cancer cell lines depleted from individual AKT isoforms on cell viability. A. Graphs represent the results of MTT assay performed on **A.** OVCAR3sfRon-shAKT1, **B.** OVCAR3sfRon-shAKT2, **C.** OVCAR4-shAKT1 and **D.** OVCAR4-shAKT2 cell lines treated with AD80 and/or BMS777607. The results show percentage increase in dead cells of drug-treated cells vs. control cells (vehicle) that were assumed to be 100% viable (0% of dead cells). ***A*-*F*.** was established by using unpaired t test. The following symbols indicate the statistical significance of data: NS = not significant, * = $p < 0.05$, ** = $p < 0.01$, *** = $p < 0.001$ and **** = $p < 0.0001$.

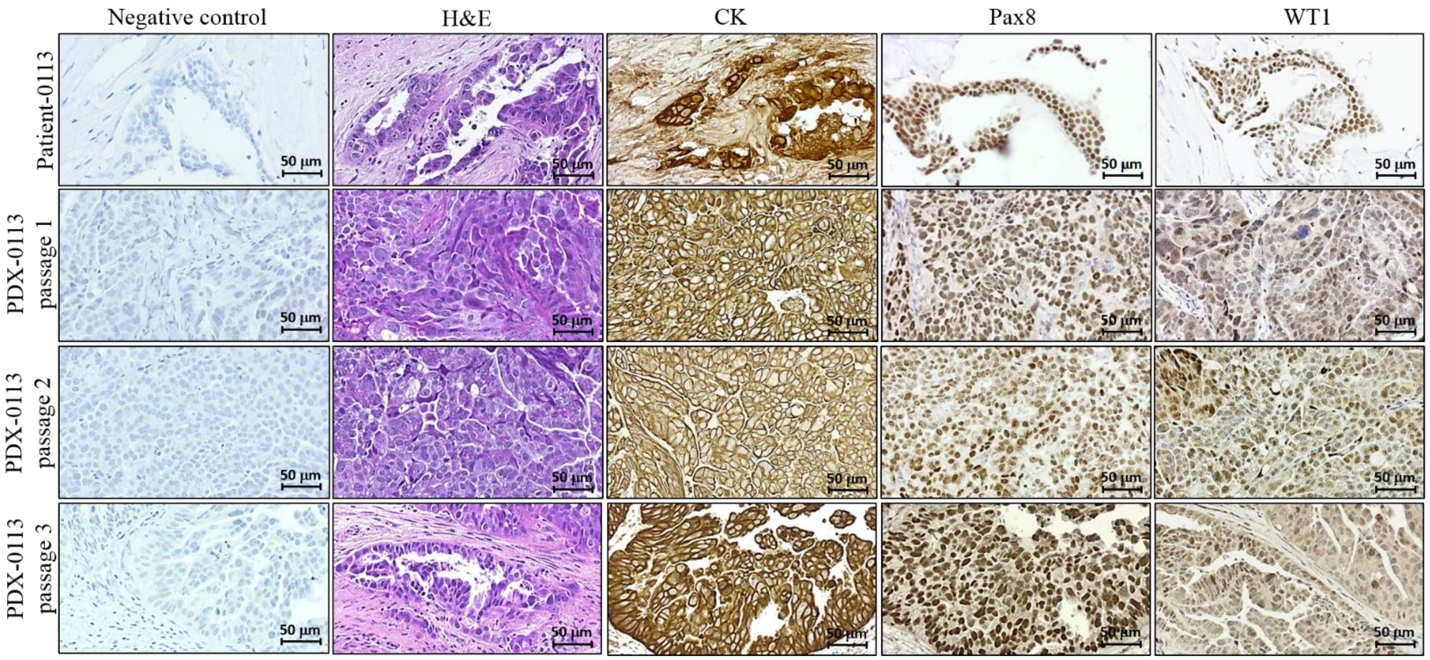

Supplementary Figure 5: Generation of patient-derived tumor models from high-grade serous ovarian cancer (HG-SOC) samples. Patients' tumors were implanted orthotopically (via the intra-ovarian bursa, IB) into immune-deficient NOD/SCID mice, and then serially passaged for three generations. Photographs show histological and immunohistochemical characteristics of high-grade serous ovarian tumor (# 0113) from patient compared with three consecutive passages of its derivative PDX-0113 line. Histology of the original tumor as well as the expression of commonly used markers for HG-SOC such as pan-cytokeratin (CK), PAX8 and WT1 are maintained in PDX lines.

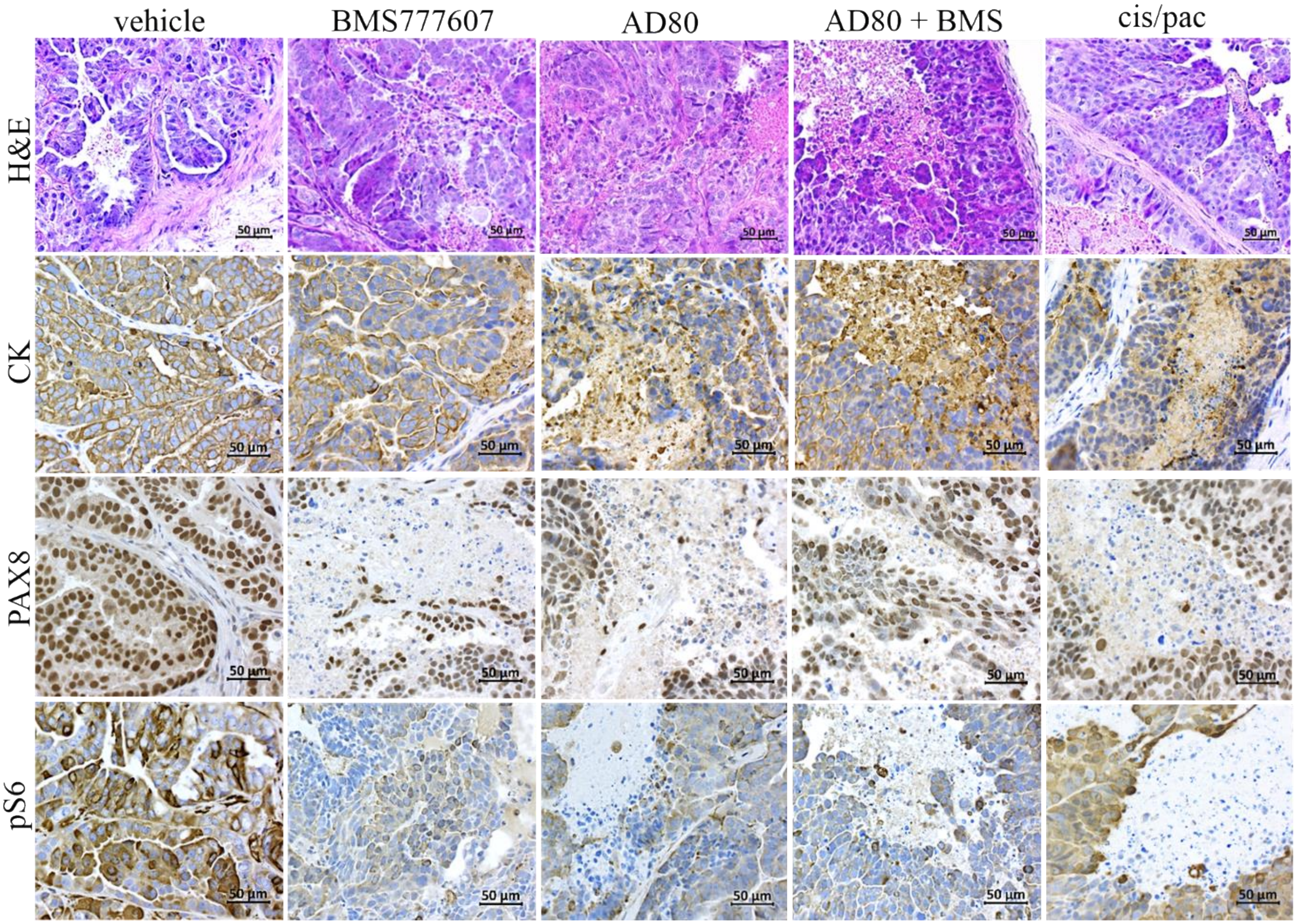

Supplementary Figure 6: Representative sections of tumors obtained from the therapeutic experiment performed on PDX-0113 treated with different drug regimens (described in Fig. 5B, C in main manuscript). Tumor sections were H&E stained and immunohistochemically evaluated for pan-cytokeratin (CK), PAX8 and pS6. The expression of pan-cytokeratin (CK) and PAX8 is maintained in PDX tumor sections representing each treatment modality. The expression of phosphorylated ribosomal protein S6 (pS6) is decreased in tumors treated with sfRon pathway inhibitors such as BMS777607 and/or AD80, but not in tumors treated with cisplatin/paclitaxel (cis/pac) chemotherapy, as expected.

A

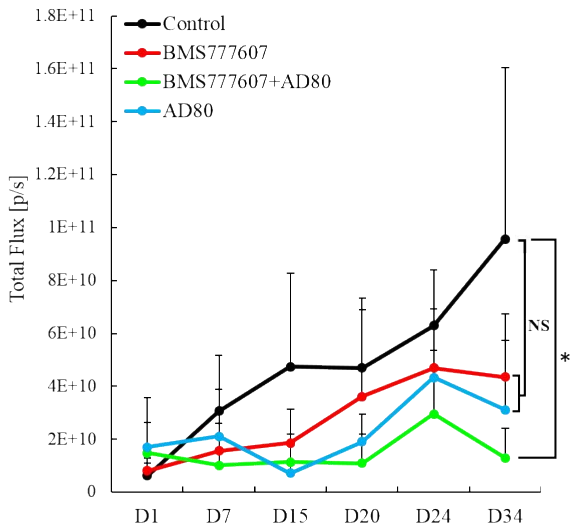

B

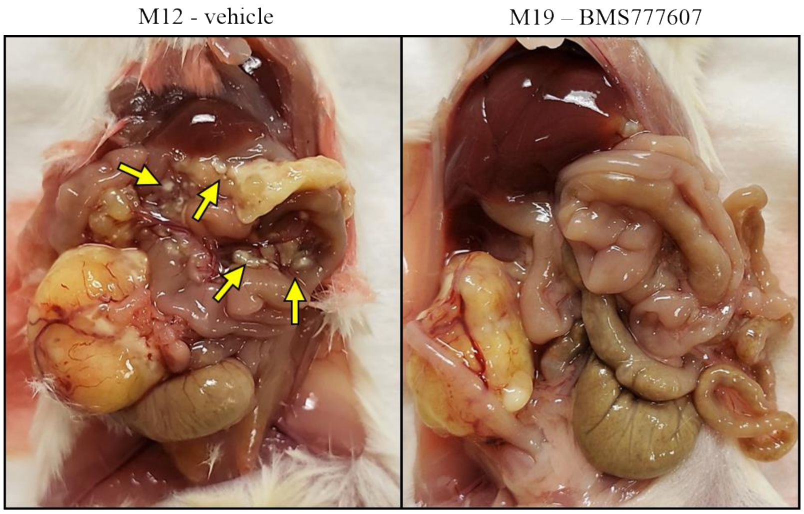

C

Assessment of metastasis		
**Treatment**	**Number of mice**	**% of incidence**
Control	4/5	80%
BMS777607	0/5	0%
BMS + AD80	0/5	0%
AD80	0/5	0%

Supplementary Figure 7: A. NOD/SCID mice were injected with luciferized OVCAR4 cells and treated with AD80 +/- BMS777607 treatment regimens as shown in Figure 4 in the main manuscript. Luciferase expression was measured weekly and quantified at day 34 using the LivingImage analysis software (Xenogen). Statistical significance of data was assessed by one way ANOVA analysis (NS = not significant, * = p < 0.05). **B.** Representative images of mice with and without ovarian tumor metastasis. Yellow arrows point to metastatic lesions in the peritoneal cavity of vehicle treated mouse, while BMS777607 treated mouse shows no detectable metastasis. **C.** Frequency of metastases across different treatment groups is represented in the table. At necropsy, 80% of the control mice showed multiple metastatic lesions, while mice treated with targeted therapies have only primary tumor without detectable metastases.

Supplementary Table 1: Quantification data of a phosphorylation kinetics of proteins within sfRon pathway. OVCAR3sfRon cells were exposed to different treatments described in **Fig. 3E** in main manuscript. Proteins were separate by WES and quantified using Compass for Simple Western software and represented as percentage change in the expression of selected phosphorylated proteins in each drug treatment condition, assuming 100% protein expression in control (vehicle-treated) cells.

	**2h treatment**					**2h washout**					**4h washout**				
**% change of protein expr.**	**Veh**	**BMS**	**BKM**	**AD80**	**BMS+ AD80**	**Veh**	**BMS**	**BKM**	**AD80**	**BMS+ AD80**	**Veh**	**BMS**	**BKM**	**AD80**	**BMS+ AD80**
p-sfRon	100	0	92	70	0	100	44	101	95	0	100	88	121	92	52
$pAKT^{S473}$	100	19	2	1	1	100	29	21	1	0	100	27	25	0	1
$p4E\text{-}BP1^{T37/46}$	100	79	63	47	62	100	107	127	37	47	100	122	125	31	26
$pS6K1^{T389}$	100	29	26	189	158	100	79	122	57	71	100	158	192	101	76
